# Sex-Specific Variation of Social Play in Wild Immature Tibetan Macaques, *Macaca thibetana*

**DOI:** 10.3390/ani11030805

**Published:** 2021-03-13

**Authors:** Tong Wang, Xi Wang, Paul A. Garber, Bing-Hua Sun, Lixing Sun, Dong-Po Xia, Jin-Hua Li

**Affiliations:** 1 School of Life Sciences, Anhui University, Hefei 230601, China; wangtong19930604@163.com; 2 International Collaborative Research Center for Huangshan Biodiversity and Tibetan Macaque Behavioral Ecology, Hefei 230601, China; wangxi198307@163.com (X.W.); Binghuasun00@126.com (B.-H.S.); 3 School of Resource and Environmental Engineering, Anhui University, Hefei 230601, China; 4 Department of Anthropology and Program in Ecology, Evolution, and Conservation Biology, University of Illinois, Urbana, IL 61801, USA; p-garber@illinois.edu; 5 International Centre of Biodiversity and Primate Conservation, Dali University, Dali 671000, China; 6 Department of Biology, Central Washington University, Ellensburg, WA 98926, USA; lixing@cwu.edu

**Keywords:** *Macaca thibetana*, social play, aggression, grooming

## Abstract

**Simple Summary:**

Social play among immature individuals has been well-documented across a wide range of mammalian species. It represents a substantial part of the daily behavioral repertoire during immature periods, and it is essential for acquiring an appropriate set of motor, cognitive, and social skills. In this study, we found that infant Tibetan macaques (*Macaca thibetana*) exhibited similar patterns of social play between males and females, juvenile males engaged more aggressive play than juvenile females, and juvenile females engaged more affiliative play than juvenile males. Our results provided more evidence to understand the functional differences of social play in immature nonhuman primates.

**Abstract:**

Theories proposed to explain social play have centered on its function in establishing social relationships critical for adulthood, its function in developing motor skills needed to survive, and promoting cognitive development and social learning. In this study, we compared variations in social play among infant and juvenile male and female *Macaca thibetana*. Given that this species is characterized by female philopatry and male dispersal, we hypothesized that immature females use social play as a mechanism to develop bonds that persist through adulthood whereas immature males use play to develop social skills needed to successfully enter new groups. The results indicated that social play steadily increased during the infant period and peaked at approximately 12 months of age. There were no significant differences in the frequency or types of social play exhibited between infant males and infant females. During the juvenile period, however, social play was found to decrease with age, with males engaging in social play more frequently than juvenile females. Moreover, whereas juvenile males engaged in more aggressive forms of play, juvenile females engaged in more affiliative forms of play. In addition, juvenile females engaged in higher rates of grooming than juvenile males. These results provide evidence of sex-specific differences and imply the functional variation of social play in Tibetan macaques, with immature males using social play to develop skills needed to enter and enhanced their dominance rank in a new social group and immature females using social play to develop long-term same-sex social bonds in their natal group.

## 1. Introduction

Play represents a substantial part of the daily behavioral repertoire during infant and juvenile development in social mammals, and it is essential for acquiring an appropriate set of motor, cognitive, and social skills [1,2,3]. For example, in meerkats [4], yellow-bellied marmots (*Marmota flaviventris*) [5], wolves (*Canis lupus*) [6], dogs (*Canis familiaris*) [7], and several species of nonhuman primates [8,9,10,11], play accounts for up to 18% of the infant and 10% of the juvenile daily activity budget. Depending on the species, play can occur in a variety of forms and contexts, including solitary or self-directed play, play with objects in the environment, or play with one or more conspecifics (social play) [12].

For many animal species, the set of factors that drive social play, its immediate and long-term benefits, and the social and ecological conditions in which social play occurs are not well-understood [13]. Burghardt proposed that social play is recognized by the following characteristics: it does not contribute to immediate survival needs, it is spontaneous, it is voluntary, it is repetitive, it is not stereotypic, and it is performed when individuals are in a relaxed state [2]. In addition, social play differs from the majority of behaviors exhibited by adults such as allogrooming, huddling, forms of aggressive interactions, and copulatory behaviors (see Palagi) [14] in form (social play is often exaggerated), timing (the set of behaviors during social play often change rapidly, and may reemerge several times during the same play sequence) [15], and the threat of injury (individuals are rarely harmed during play bouts).

A major theoretical question in the study of social play is whether the variations are specific to the developmental period in which the behaviors occur (e.g., during the infant and juvenile periods) or whether the ultimate function of play is the development of social, cognitive, and motor skills, as well as the formation of social relationships required for life as an adult [5,15]. In Assamese macaques (*Macaca assamensis*). Berghänel et al. found that immature males participated more in locomotor play than immature females, and this resulted in males acquiring motor skills at an earlier age than females [9]. The idea that juvenile play functions in developing skills needed for adult survivorship was supported in a study of yellow-bellied marmots (*Marmota flaviventris*) [5].

It has also been argued that a positive relationship exists between the frequency of social play and cognitive development [16,17]. For example, Lewis examined the types and frequency of play and the neocortex ratio (size of the neocortex relative to the size of the entire brain) of seven species of immature, captive, nonhuman primates [3]. The neocortex represents a part of the brain associated with higher-order cognitive function. She found no relationship between the neocortex ratio and the frequency of solitary forms of play (locomotor or object play). However, social play was positively correlated with the neocortex ratio, suggesting an important relationship between evolutionary changes in the ability of the brain to process complex social information and a behavioral pattern of practicing social skills during infant and juvenile development [3]. Moreover, the social intelligence hypothesis predicts that increased social play enables individuals to more effectively develop flexible motor, behavioral, and emotional responses to unexpected events [1]. For example, in a study of captive vervet monkeys (*Chlorocebus aethiops*), Fedigan found that both infants and juveniles preferentially selected particular playmates and the type of play based on the anticipated reaction of that playmate to their actions [18].

Finally, in sexually dimorphic species, as well as among species that differ in patterns of residence and dispersal, males and females are expected to exhibit different ontogenetic trajectories and functions of play [19]. For example, in male philopatry and female dispersal species, philopatric males were found to exhibit a higher degree of social play than dispersal females [20]. However, in female philopatric and male dispersal species, individuals of the philopatric sex may usually be expected to use social play to develop friendships with same-sex playmates that extend throughout adulthood, whereas individuals of the dispersing sex may be expected to use social play to develop fighting abilities and social skills that can be used to establish their dominance rank and increase access to sexual partners when they enter a new group [21].

Moreover, in species characterized by female philopatry, a previous study demonstrated that maternal rank may also have a significant effect on immature social interactions and access to social partners [18]. For example, it has been suggested that females of high-ranking matrilines might increase their reproductive success by preferentially investing in daughters, who will remain in their natal group and assist their mothers and close relatives [22]. In contrast, low ranking mothers might preferentially invest in the competitive ability of their sons, who will attempt to establish high rank in a neighboring group [23].

In this study, we examined social play in wild Tibetan macaques, *Macaca thibetana*, a near-threatened species of nonhuman primate. Tibetan macaques are endemic to China, with a remaining population size of some 20,000 individuals [24]. Tibetan macaques form a multi-male/multi-female social group, which on average contains nine adult males, ten adult females, 15 juveniles, and five infants (data from the Yulinkeng A1 (YA1) group between 1987 and 2019). In this sexually dimorphic species, adult males weigh approximately 16.4 kg and adult females weigh approximately 11.0 kg [24]. Males attain sexual maturity by age seven, whereas females reach sexual maturity by age five. In Tibetan macaques, females are philopatric and form long-term and stable social relationships with female relatives [24]. In contrast, males emigrate from their natal group at six-to-seven years of age and form bonds with resident females in an attempt to integrate into the new group [24]. Specifically, we compared variation in the percentages of aggressive/affiliative play, the number of play partners, and play rates in infant and juvenile male and female Tibetan macaques. We also examined patterns of affiliative and agonistic behavior in adult male and female Tibetan macaques to provide further evidence on the sex-specific variations of social play during their immature periods. We used these data to test the following hypotheses.

**Hypothesis** **1** **(H1).** *Assuming that play has an immediate function in developing the social and motor skills needed by both males and females to survive the infant period (0–12 months of age), we expect no differences in the patterns and frequency of social play (i.e., affiliative play and aggressive play) between infant male and infant female Tibetan macaques.*

**Hypothesis** **2** **(H2).** *In Tibetan macaque society, females are philopatric and are required to establish their social bonds with group members in their natal group, whereas males disperse and are required to establish their social position in their new group. Assuming that social play during the juvenile period (12–60 months of age for females and 12–84 months of age for males) functions to develop social relationships required for adulthood, we expect the philopatric sex (i.e., females) to more frequently engage in affiliative and less aggressive forms of social play, whereas juveniles of the dispersing sex (i.e., males) are expected to more frequently engage in aggressive and less affiliative forms of social play. If so, we would also expect adult female intrasexual social interactions to be more affiliative and less aggressive than adult male intrasexual social interactions.*

**Hypothesis** **3** **(H3).** *Assuming that social play in infants and juveniles is affected by maternal rank and/or matriline affiliation/rank, we expect both male and female offspring of higher ranking mothers to engage in more frequent bouts of play and with a larger number of social partners than the offspring of lower ranking mothers.*

## 2. Materials and Methods

### 2.1. Study Site and Subjects

We conducted this study at the Mt. Huangshan National Reserve in Anhui Province, China. The study site is located within the reserve in an area known as the “Valley of the Monkeys.” For more details of the study site, see the work of Xia et al. [25]. The study group was the YA1 group. Demographic data have been collected on a daily basis on this population since 1986 [26], and matrilineal kin relationships are known for all female members of our study group. All adult members of the study group are individually recognizable based on their distinctive physical features (e.g., scars, hair color patterns, and facial/body features).

The study group is habituated to researchers (i.e., from <1 m) and was provisioned daily with 3–4 kg of corn (approximately 60 g/individual) by reserve staff to maintain their presence at designated tourist-viewing sites during 2004 and 2014. Though there have been no tourists since 2014, the study group was still provisioned by reserve staff in the same manner. After feeding, the monkeys leave the provisioned area and continue their natural and undisturbed activities in the forest.

Our study subjects were the 61 individuals of the YA1 group, including 30 adults (10 males and 20 females), 12 juveniles (seven males and five females), and 19 infants (nine males and ten females) (Table 1). Adult males were >7 years of age, adult females were >5 years of age, juvenile males were 1–7 years of age, juvenile females were 1–5 years of age, and infants were individuals <1 year of age. Age categories were from the work of Li et al. [24]. Except for one group member, PY, an immature male, all of the immature individuals were members of three matrilines, T, H or Y. The first letter of the ID of an immature indicates its matriline affiliation (see Table 1).

### 2.2. Data Collection

We collected behavioral data from January to June 2019 (totaling 131 days, mean ± SE = 21.83 ± 0.26 days/month, range = 19–24). Following the protocol used by Xia et al. [25,26], we followed the group from dawn to dusk, with behavioral observations beginning at approximately 07:00–08:00 and ending at 17:00–18:00 each day, depending on the time of year. We collected data from a distance of 5–10 m. We used a digital voice recorder (Lenovo Recorder; model: B618) or a digital video camera (Sony Digital HD Camcorder; model: HDR-PJ50E) to record the activity of the monkeys. We collected all the data when the monkeys were in the natural forest, without the influence of human activity.

We used a continuous focal animal sampling method to collect data on all the daily behaviors of a target individual, including social play, allogrooming, agonistic behavior, and other daily activities. We set each focal sampling period as 20 min, according to Xia et al. [27]. If the focal monkey could not be followed or was lost from view during the sampling period, another individual was randomly selected [24]. An effort was made to locate and record the behavior of the lost individual during the next 20 min sampling period [27]. Focal sampling yielded a total of 214.48 h of data (mean ± SE = 3.52 ± 0.110 h; *n* = 61; range: 0.03–4.51 total h per monkey).

We needed to know the percentage of immature individuals participating in different types of social play and to judge the change in the play style of different individuals from this. However, due to the short duration of play and rapid changes, it was very difficult to continuously record each type of play. Considering the fact that the data of play were not as large as expected, we used the sampling methods of Shimada and Sueur [10]. During the focal sampling periods, we divided each 20 minute period into 20 one-minute units and used a one-zero sampling method [26] to record the presence/absence and types of social play among all individuals within a 3-m radius of the focal individual [10]. We recorded the play type that first appeared in each focal block. A radius of 3 m was selected because that coincided with the area contained in the video we recorded.

According to Burghardt’s identification criteria of social play, it was feasible to recognize the difference between social play and non-play behaviors. In this study, we defined social play as behaviors functioned to develop, practice, or maintain physical or cognitive abilities and social relationships, including both tactics and strategies, by varying, repeating, and/or recombining already functional sub-sequences of behavior outside their primary context [14]. We categorized them into aggressive playing as rough pattern and affiliative playing as soft pattern. More detail definitions, modified from the work of Wright et al. and Pal [7,28], can be found in Table 2. In total, we scored 7 different play behaviors. A play bout began when two or more individuals came into direct contact and engaged in chasing or other play behaviors (Table 2). A play bout ended when all players initiated non-play activities (e.g., resting, social grooming, and travel), withdrew from the bout, or adult interference resulted in all players ceasing their play activities [29].

We restricted our analysis to social play that involved only two players (dyadic social play). This was done to avoid repeated calculations of the same data (e.g., A chasing B, A chasing C, and B chasing C), as suggested in Shimada and Sueur [10]. Thus, dyadic social play involved two individuals (e.g., individual A and B) and included two events (individual A plays with individual B and individual B plays with individual A). We next analyzed the data matrices of social play obtained from our two different sampling methods (focal sampling method and one-zero method). The results indicated statistically similar patterns (Matrix correlation test: Kr = 1448, Pr < 0.001, based on 2000 permutations; see below under Data Analysis) [30,31]. Therefore, we combined these two datasets to test Hypotheses 1 and 2.

Though social play can be easily recognized according to Burghardt’s criterion [2], social grooming remains difficult to be categorized as either a form of social play or as a form of non-play social behavior. In this study, we categorized grooming between immature individuals as a non-play social behavior. We used a continuous recording method (in seconds) to quantify the duration of grooming bouts between two individuals of all age/sex classes. Grooming was defined as any act in which a macaque (groomer) used its hand or mouth to touch, clean, or manipulate the fur of another individual (groomee) for a continuous period lasting at least 5 s [25,26,27].

Data were also collected on adult aggressive and submissive behaviors using a focal animal sampling. We used a continuous recording method to collect agonistic behaviors. Aggression was defined as an individual threatening, chasing, slapping, grabbing, or biting another individual [32]. Submissive behavior was scored when an individual exhibited a fearful response, such as fear grin, cower, mock leave, avoid, flee, or scream [32]. These data were used to test Hypothesis 2.

In addition, we used an *ad libitum* sampling method to collect aggressive/submission interactions. We used these data to build an aggressive/submission matrix, calculate an individual’s normalized David’s score (NDS; Table 1), and determine the individual social rank of adults (see [25]). The greater an individual’s NDS, the higher its social rank. Based on individual NDS values, we used a k-means cluster analysis (100 iterations) to classify each adult female into high-, middle-, and low-ranking dominance classes [30]. Given that a female’s rank is largely determined by the rank of her mother and close female relatives, we calculated matriline rank. The study group was composed of three matrilines. Y, H, and T matriline affiliation was based on 30 years of genealogical data on the YA1 group. Matriline rank was scored as the average NDS of adult females residing in a given matriline. Y was the highest ranking matriline (mean NDS = 17.06 ± 48.82), H was the middle ranking matriline (mean NDS = −12.75 ± 32.25), and T was the lowest ranked matriline (mean NDS = −80.18 ± 22.09). We found a significant correlation between maternal rank and matriline rank (*r*s = 0.725; *n* = 148; *p* < 0.001).

### 2.3. Data Analysis

We used a row-wise correlation test to examine the correlation matrices between infant and juvenile social play obtained using the focal sampling method and the one-zero method (Kr test) [30]. Hemelrijk’s MatrixTester v2.23 program was used for these computations [31]. The results were based on 2000 simulations.

Because maternal rank can affect immature and adult social interactions in Tibetan macaques [24], it was necessary to control for female rank to test Hypotheses 1, 2, and 3. We report data as mean (±SE) for the rate of play (episodes/h), the percentage of aggressive and affiliative interactions during social play, the rate of grooming (bouts/h within dyads), and the rate of aggression (episodes/h only for adults). We used a one sample Kolmogorov–Smirnov test to examine whether the data conformed to a normal distribution (*p* > 0.05).

To clarify how the individual attributes influenced the extent of social play, we carried out multiple regression analysis, in which the rate of social play and playmates number represented response variables, while age (month), sex (male and female), maternal rank (high, middle, and low), matriline affiliation (Y, H, and T)/rank (high, middle, and low) represented explanatory variables. We controlled individual IDs as the control variable in the model. We ran those models for infants and juveniles.

We used a Wilcoxon signed rank test to determine differences in the number of social play bouts between infant males, infant females, juvenile males, and juvenile females. We also used a Wilcoxon signed rank test for the analysis of differences in the types of play engaged in by infant males, infant females, juvenile males, and juvenile females. We compared the percentage of aggressive play and affiliative play between infants and juveniles. We used the Wilcoxon signed rank test to assess differences in the rate at which males and females of different age classes initiated intrasexual aggressive behavior and intrasexual social grooming.

All analyses, unless specified, were two-tailed and were carried out using the SPSS 22.0 software (SPSS Inc., Chicago, IL, USA), with the significance level set a priori at 0.05.

## 3. Results

During the study period, 2343 dyadic social play bouts among 31 immature individuals (*n* = 19 males and 12 females) were recorded. Infants engaged in 3040 (infant males: 2355; infant females: 685) play bouts, and juveniles engaged in 1646 (juvenile males: 1340; juvenile females: 306) play bouts. Males engaged in significantly more bouts of social play than females (male: 39.78 ± 5.25 bouts; female: 16.16 ± 3.97 bouts; Wilcoxon signed rank test: *Z* = −2.456; *p* = 0.014). Among all play interactions, 2017 (86.1%) were defined as aggressive, and 326 (13.9%) were defined as affiliative. In the case of infants, 91.5% of play was aggressive and 8.5% was affiliative. For juveniles, 69.4% of play was aggressive and 30.6% was affiliative.

The percentage of social play in the daily activity budget of infant males was 5.3% and for infant females, it was 2.1%. In the case of juveniles, the proportion of the activity budget devoted to social play was 3.6% for males and 1.5% for females. Overall, both males and females ceased social play as adults (this occurred at 6.67 years of age in males and at 4.17 years of age in females,).

### 3.1. Testing H1: Sex-Based Differences in Infant Social Play

**Figure 1:**
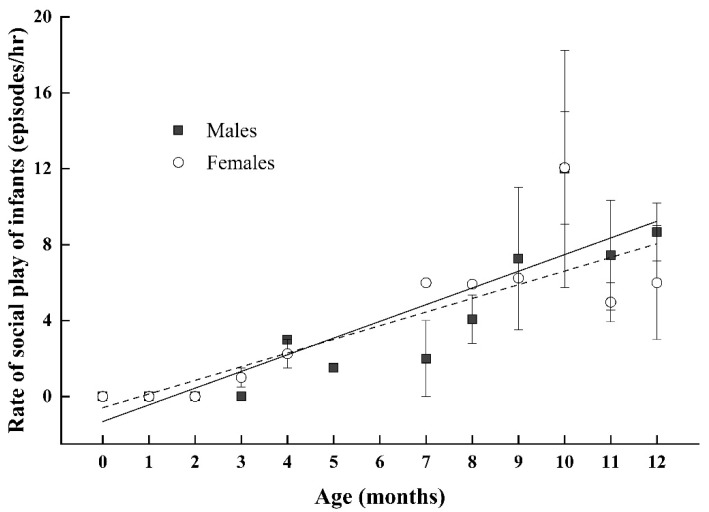
Correlation between the rate of social play and age in infant male (black square) and infant female Tibetan macaques (white circle). Each point may represent multiple individuals depending on the number of individuals of certain month ages. The solid and dashed lines indicate the estimated trend for infant males and infant females, respectively.

As indicated in Figure 1, during the first three months of life, infant Tibetan macaques rarely participated in bouts of social play (0.083 bouts/h). However, by six months of age, social play in infants had increased to 3.332 bouts/h. During the first 12 months of life, both males and females exhibited a similar positive increase in the frequency of social play with age (infant males: *β* = 0.470, *p* = 0.004; infant females: *β* = 0.744, *p* < 0.001; results from multiple linear regression model:R^2^ = 0.423, F4, 31 = 7.421, *p* < 0.001 for infant males; R^2^ = 0.720, F4, 21 = 17.076, *p* < 0.001 for infant females; see Figure 1). The results showed that there was no significant difference in the rate of play between infant males and females (infant males: 2.77 ± 0.86 episodes/h; infant females: 2.30 ± 0.73 episodes/h; *Z* = −0.087; *p* = 0.931).

**Figure 2:**
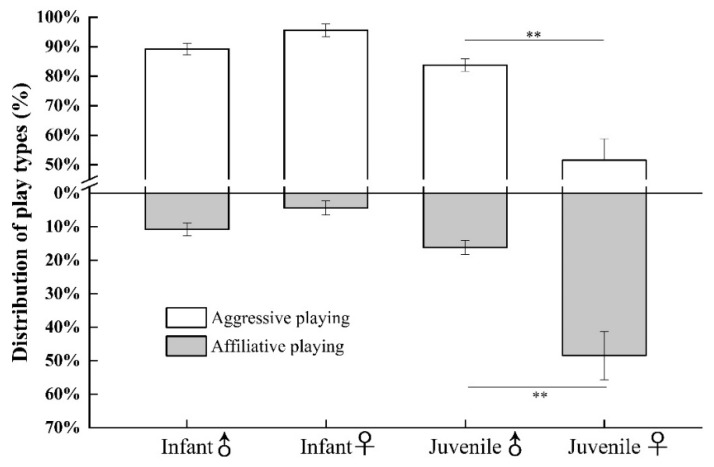
Variation in the percentage of aggressive and affiliative play in infant male, infant female, juvenile male, and juvenile female Tibetan macaques. (** *p* < 0.01).

As indicated in Figure 2, there was no significant difference in the percentage of aggressive play bouts engaged in by infant males and infant females (infant males: 89.24% ± 1.88%; infant females: 95.63% ± 2.12%, *Z* = −1.792, and *p* = 0.073). Similarly, the frequency of affiliative play did not differ by sex (infant males: 10.76% ± 1.88%; infant females: 4.37% ± 2.12%, *Z* = −1.792, and *p* = 0.073).

### 3.2. Testing H2: Sex-Based Differences in Juvenile Social Play

**Figure 3:**
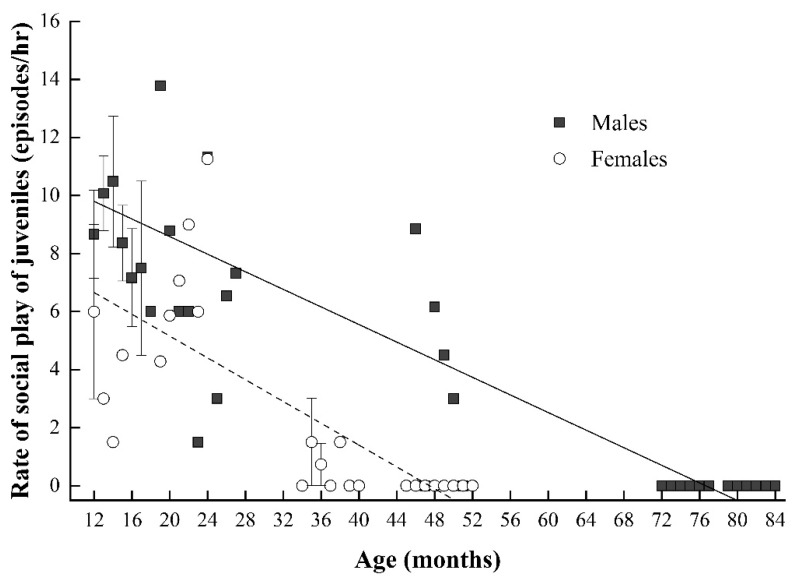
Correlation between the rate of social play and age in juvenile male (black square) and female Tibetan macaques (white circle). Each point may represent multiple individuals depending on the number of individuals of certain month ages. The solid and dashed lines indicate the estimated trend for juvenile males and juvenile females, respectively.

We found that during the juvenile period, both males and females were characterized by a negative correlation between the rate of social play and age through a multiple linear regression model (juvenile males: *β* = −0.839 and *p* < 0.001 based on multiple linear regression model: R^2^ = 0.647, F4, 46 = 23.869, and *p* < 0.001; juvenile females: *β* = −1.123 and *p* < 0.001 based on multiple linear regression model: R^2^ = 0.708, F4, 30 = 21.617, and *p* < 0.001; see Figure 3).

There were significant sex-based differences in the frequency of social play, with juvenile males playing more than juvenile females (juvenile males: 5.43 ± 0.64 episodes/h; juvenile females: 2.06 ± 0.53 episodes/h, *Z* = −4.642, and *p* < 0.001). Though the majority of play in both juvenile males and females was aggressive, the percentage of aggressive play in males (83.80% ± 2.10%) was significantly greater than that of females (51.57% ± 7.20%, *Z* = −3.343, and *p* = 0.001). Relatedly, the percentage of affiliative play in juvenile females (48.43% ± 7.20%) was significantly greater than that of juvenile males (16.20% ± 2.10%; see Figure 2). These data offer support for H2 (i.e., social play in juvenile females was less aggressive and more affiliative than in juvenile males).

### 3.3. Testing H2: Sex-Based Differences in Social Interactions

**Figure 4:**
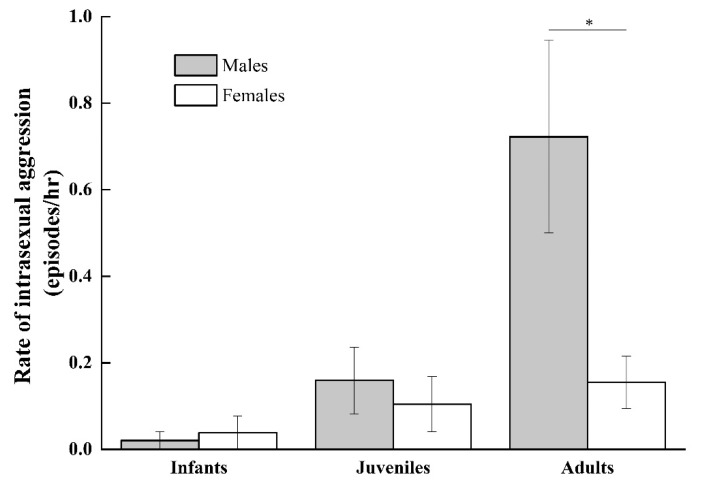
Rate of aggressive interactions between infant males, infant females, juvenile males, juvenile females, adult males, and adult females. (* *p* < 0.05).

**Figure 5:**
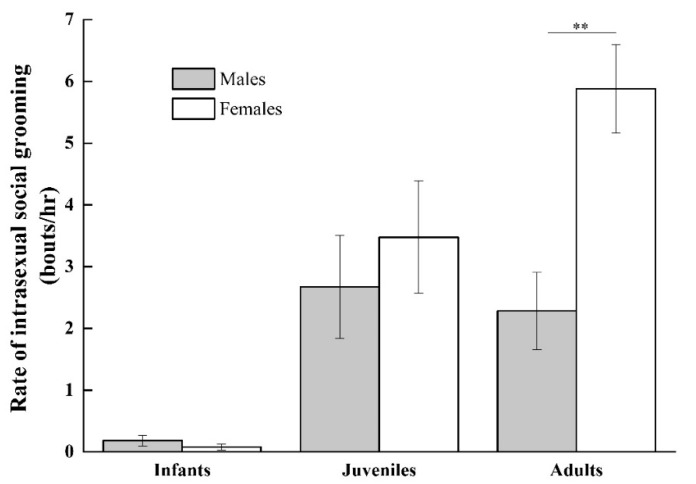
Rate of grooming interactions between infant males, infant females, juvenile males, juvenile females, adult males, and adult females. (** *p* < 0.01).

As indicated in Figure 4 the rate of intrasexual aggressive social interactions that occurred outside the context of play increased with age but was nevertheless relatively low among infants (infant males: 0.02 ± 0.02; infant females: 0.04 ± 0.04) and juveniles (juvenile males: 0.07 ± 0.07; juvenile females: 0.05 ± 0.005) and did not differ by sex (infant: *Z* = −0.447 and *p* = 0.655; juvenile: *Z* = −0.447 and *p* = 0.655). Similarly, the rate of intrasexual grooming increased with age and did not differ between infant males and females or between juvenile males and females (infant males: 0.06 ± 0.05; infant females: 0.08 ± 0.05, *Z* = −1.069, and *p* = 0.285; juvenile males: 1.85 ± 0.59; juvenile females: 1.44 ± 0.29, *Z* = −0.674, and *p* = 0.500; see Figure 5).

A comparison of adult intrasexual social interactions indicated that adult males more frequently engaged in aggressive interactions than adult females (adult males: 0.61 ± 0.19 episodes per hour; adult females: 0.15 ± 0.06 episodes per hour, *Z* = −2.028, and *p* = 0.043). Adult females engaged in higher rates of intrasexual grooming than adult males (adult females: 3.48 ± 0.42 bouts per hour; adult males: 0.68 ± 0.20 bouts per hour, *Z* = −2.803, and *p* = 0.005). Thus adult female intrasexual social interactions were overall more affiliative and less aggressive than adult male intrasexual social interactions.

### 3.4. Testing H3: Rate of Play and Number of Playmates

**Figure 6:**
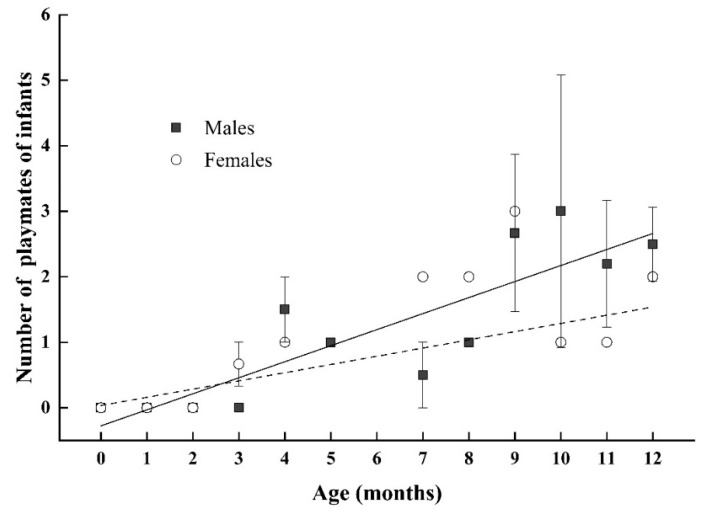
Correlation between the number of playmates and age in infant male (black square) and infant female Tibetan macaques (white circle). Each point may represent multiple individuals depending on the number of individuals of certain month ages. The solid and dashed lines indicate the estimated trend for infant males and infant females, respectively.

For infant Tibetan macaques, we did not find a correlation between the rate of social play and mother’s rank. In addition, there was no significant correlation between the rate of play and matriline affiliation/rank in infants of either sex (Table 3). Though we did find that although the number of playmates increased with age (infant males: *β* = 0.506 and *p* = 0.003; infant females: *β* = 0.751 and *p* < 0.001; Figure 6), there was no significant effect of maternal rank or matriline affiliation/rank on the number of playmates infant males or infant females played with (results from multiple linear regression model—infant males: R^2^ = 0.386, F4, 31 = 6.509, and *p* = 0.001; infant females: R^2^ = 0.558, F4, 21 = 8.888, and *p* < 0.001; Table 3).

**Figure 7:**
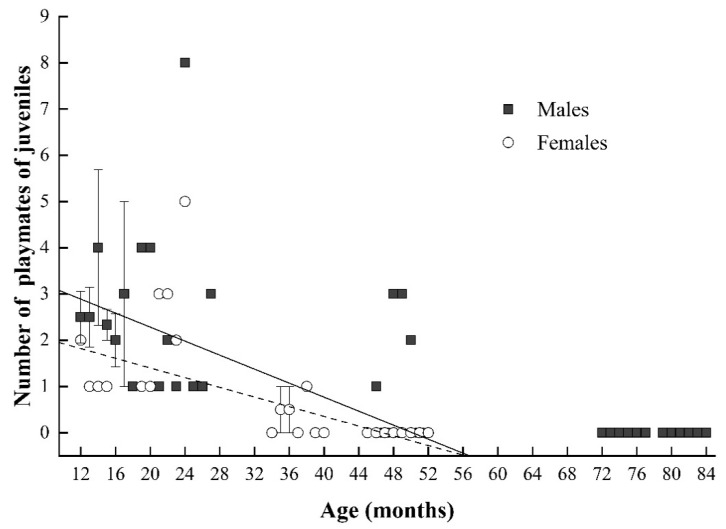
Correlation between the number of playmates and age in juvenile male (black square) and female Tibetan macaques (white circle). Each point may represent multiple individuals depending on the number of individuals of certain month ages. The solid and dashed lines indicate the estimated trend for juvenile males and juvenile females, respectively.

For juvenile Tibetan macaques, males and females also exhibited a significant positive relationship between the rate of social play (bouts per hour) and mother’s rank. However, matriline affiliation/rank had no significant effect on the rate of social playing in either juvenile males or females (Table 4). We also found evidence of a negative correlation between the number of playmates and age in juvenile males and females (juvenile males: *β* = −0.538 and *p* = 0.001; juvenile females: *β* = −0.870 and *p* < 0.001; Figure 7). Thus, it appears that as juveniles mature, they concentrate their play activities on a limited set of play partners. The number of playmates, however, was not correlated with maternal rank or matriline affiliation/rank in either juvenile male or female Tibetan macaques (results from multiple linear regression model—juvenile males: R^2^ = 0.273, F4, 46 = 5.705, and *p* = 0.001; juvenile females: R^2^ = 0.477, F4, 30 = 8.758, and *p* < 0.001; Table 4).

## 4. Discussion

Play is a critical behavior required for the development of appropriate cognitive, physical, and social skills in many animal species [2,33,34]. In addition, as suggested by Paukner and Suomi and Blumstein et al. [5,19], an understanding of sex-based differences in social play during the infant and juvenile periods offers important insight into sex-based differences in adult social interactions and social relationships. Compared to many mammalian lineages, primates are characterized by an extended period of infant and juvenile development, during which individuals obtain critical social and ecological skills through a combination of trial-and-error learning, various mechanisms of social learning, and a process of innovative decision-making [35]. Though young primates can learn social skills by engaging with any group member, social play among similar aged individuals is likely to play a particularly important role in the ontogeny of social cognition [3]. In this study, we examined evidence for age- and sex-based differences in social play in wild infant and juvenile Tibetan macaques, a female philopatric species.

According to the motor-training hypothesis, the primary function of play in immature primates is to practice and develop motor skills with minimal risk of injury [33,36]. Assuming this to be the case, we hypothesized that infant male and infant female Tibetan macaques would exhibit similar patterns of play and social interactions early in development (H1). Our data confirmed the predictions for this hypothesis—as the rate of social play increased with age during infancy (age 0–12 months), both males and females engaged in similar forms of social play and at relatively similar frequencies. In addition, during the infant period, neither maternal rank nor matriline affiliation/rank had a significant effect on social play in males or females. Our results differed from studies of wild chimpanzees (*Pan troglodytes*), captive patas monkeys (*Erythrocebus patas*), captive Japanese macaques (*Macaca fuscata*), and captive rhesus macaques (*Macaca mulatta*), which found that infant males were characterized by an increase in the amount of social play compared to infant females [37,38,39,40]. Similarly, Brown and Dixon documented the presence of sex-based differences in play behavior during the first few months of infancy in captive rhesus macaques (*Macaca mulatta*) [39], with males exhibiting more frequent play and higher rates of “rough-and-tumble play and mounting than female infants, and also exhibited stationary play, chasing play, and initiated play more frequently than females.”

Relatedly, although we did not find evidence of that maternal rank affected the frequency of social play in wild infant Tibetan macaques, maternal rank was found to have a significant effect on mother–infant interactions in captive rhesus macaques. In this species, the infants of high ranking and medium ranking mothers engaged in less frequent social play than the infants of low ranking mothers [39]. Differences in patterns of social play across macaque species may reflect species-specific differences in dominance style, with some macaque species characterized by high levels of aggression and a rigid linear dominance hierarchy (e.g., *Macaca mulatta* or *Macaca fuscata*), whereas other species are characterized by increased social tolerance, conciliatory behavior, and a more relaxed social style (e.g., *Macaca tonkeana* or *Macaca nigra*) [41,42]. Tibetan macaques were categorized into despotic society with the rhesus macaque, and differences in social play between infant Tibetan macaques and captive rhesus macaques also could reflect intrinsic differences in individual and maternal temperament [43].

During the juvenile period, the rate of social play among juvenile males was significantly higher than that of juvenile females. We also found that juvenile male Tibetan macaques engaged in higher rates of play and more aggressive and less affiliative forms of play than juvenile females (H2 was supported). A similar pattern of increased male aggressive play has been reported in captive Japanese macaques (*Macaca fuscata*) [41,44], captive brown capuchins (*Cebus apella*) [16], and captive and free-ranging rhesus macaques (*Macaca mulatta*) [45,46]. Moreover, a comparative study of social play in juvenile Japanese macaques and crested macaques (*Macaca nigra*) indicated that despite major differences in dominance style (Japanese macaques are highly aggressive, whereas crested macaques are among the most tolerant macaque species), juvenile males in both species engaged in longer bouts of play and more wrestling than juvenile females [42]. Petit et al. speculated that these sex-based differences are affected by the fact that juvenile females remain in closer proximity to their mothers than juvenile males and that macaque mothers are more protective of juvenile females than juvenile males [42].

In wild Tibetan macaques, we found that maternal rank had a significant effect on the frequency of social play in both juvenile males and juvenile females (H3 was supported). This was not the case during the infant period. Juveniles of higher ranking mothers (but not juveniles in higher ranking matrilines) devoted more time to social play. According to Li et al. [24], the social style of Tibetan macaques is characterized by relatively high levels of conflict and aggression. However, it remains unclear whether high ranking Tibetan macaque mothers are less protective of their juvenile offspring than lower ranking mothers or whether juvenile males and females of high ranking mothers expect their mothers to intervene in aggressive play bouts and are therefore freer to engage in higher rates of play. Given that Wright et al. reported that Tibetan macaque mothers rarely interrupt the play activities of their infant or juvenile offspring [29], it is possible that awareness of rank relationships is ‘learned’ or established early in development, and maternal interference is therefore rarely required to control the outcome of social play.

The social bonding hypothesis proposes that social relationships and rank differences formed during play among immature individuals serve critical roles in maintaining adult relationships [5,10,32,47]. Thus, we expected that in species characterized by male dispersal and female philopatry, social play during the infant and juvenile periods serves to establish female same-sex alliances, coalitions, and cooperative behaviors that continue through adulthood. In contrast, immature males were expected to engage in more aggressive forms of play that are likely to benefit them in successfully establishing residence and their dominance position in a new social group. In this regard, we found that patterns of aggressive and affiliative play during the juvenile period closely resembled patterns of aggressive and affiliative social interactions in adult Tibetan macaques. Adult female intrasexual social interactions in Tibetan macaques were more affiliative and less aggressive than adult male intrasexual social interactions. Adult females also engaged in more frequent intrasexual grooming bouts than adult males.

Our results were in line with the prediction of the social bonding hypothesis. In wild Tibetan macaques, juvenile females engaged in significantly more affiliative and less aggressive play than juvenile males. A similar pattern has been reported in captive brown capuchin monkeys (*Cebus apella*) [19] and rhesus macaques (*Macaca mulatta*) [45], both of which are all female philopatric species. In contrast, red colobus monkeys (*Procolobus rufomitratus*) are male philopatric, and juvenile males and females engaged in relatively equal amounts of aggressive play [48]. Worch argued that patterns of social play in male red colobus promote long-term social bonds among philopatric males [48].

Finally, we acknowledge that the rules governing the selection of play partners are likely to be situational. For example, to gain the most physical development from rough-and-tumble play, the ideal play partners might be individuals of similar size and strength (i.e., the same age), whereas, to facilitate long-term affiliative relationships, the ideal play partners might be close kin or individuals of the same matriline/patriline who will be close associates during adulthood [38]. In wild Tibetan macaques, however, both infant and juvenile males and juvenile females played more frequently with infant males and juvenile males and less frequently with infant females and juvenile females than expected based on the number of age/sex partners in the study group. A study of free-ranging vervet monkeys (*Chlorocebus aethiops*), a female philopatric species, also found that female infants did not preferentially select same sex play partners [36]. In the case of Tibetan macaques, other factors such as the presence or absence of sibling may have been a factor in the selection of play partners. In our study group, 9 of 19 infants had older juvenile siblings, and 9 of 12 juveniles had infant or juvenile siblings. Future studies should be conducted by controlling the availability of older siblings or matriline members to provide more evidence for more complex effects on social play in immature nonhuman primates.

## 5. Conclusions

Sex-based differences in social play in wild Tibetan macaques first emerged during the juvenile period. Juvenile male Tibetan macaques engaged in more frequent forms of aggressive playing, which appears to serve a function in developing the social and physical skills. Female Tibetan macaques are philopatric, and although they also commonly participate in aggressive forms of play as juveniles, they were also found to more frequently engage in affiliative play than males. Future studies with long-term and more data need to pay more attention to examine the effects of maternal rank and kinship on infant and juvenile play behavior and play partner choice, as both are likely to have a significant effect on sex-based differences in social behavior, social relationships, and dispersal success.

## Figures and Tables

**Table 1:** Composition of the Tibetan macaque study group. NDS: normalized David’s score.

Adult Individuals				Immature Individuals			
ID	Sex	NDS/Rank	Age (yrs)	ID	Sex	Age (yrs)	Mother
YCLO	Male		9	TXL	Male	6	TH
TRG	Male		9	PY	Male	–	–
HXM	Male		9	YXK	Male	5	YH
ZB	Male		–	TQS	Male	3	TXH
HM	Male		–	YXM	Male	>1	YCLA
DS	Male		–	TXJ	Male	>1	TH
ZF	Male		–	YQT	Male	>1	YXX
WM	Male		–	TFK	Male	<1	THY
HL	Male		–	THM	Male	<1	TRY
TQ	Male		–	TFJ	Male	<1	THX
YXX	Female	168.50/High	9	YXP	Male	<1	YCY
YH	Female	145.00/High	16	HXC	Male	<1	HH
YCY	Female	109.00/High	10	HXY	Female	3	HH
YM	Female	68.50/Middle	29	YXY	Female	3	YH
YCH	Female	51.50/Middle	7	TQY	Female	2	TXX
TXH	Female	28.50/Middle	10	TFH	Female	2	THY
HH	Female	19.50/Middle	16	TQG	Female	>1	TXH
TH	Female	4.50/Middle	16	TRJ	Female	<1	TT
TXX	Female	−25.75/Middle	11	YXT	Female	<1	YH
TQL	Female	−44.00/Middle	6	THW	Male	<1	TR
HXW	Female	−45.00/Middle	6	TQM	Male	<1	TXX
YCLA	Female	−60.50/Middle	7	THZ	Male	<1	TRX
TR	Female	−90.50/Low	15	YQX	Male	<1	YXX
THX	Female	−100.00/Low	7	YHX	Male	<1	YRL
TRY	Female	−122.00/Low	10	THS	Male	<1	TRY
TT	Female	−122.50/Low	28	TFZ	Male	<1	THY
YZ	Female	−129.00/Low	28	HQZ	Female	<1	HXW
THY	Female	−131.50/Low	10	TXD	Female	<1	TH
TRX	Female	−198.50/Low	6	TQZ	Female	<1	TXH/
YRL	Female	−216.50/Low	6	YXQ	Female	<1	YH
				YQS	Female	<1	YXY

–: Individual’s age is unknown.

**Table 2:** Definitions of play behavior.

Play Type	Behavior	Definition
Aggressive playing	Play slapping	Two animals hit each other with their hands, and their gaze is directed towards each other for a period of at least 1 s. Both sides will dodge while hitting but will not run away.
	Play wrestling	Additionally known as rough-and-tumble play. Includes play behavior in which two monkeys engage in mutual grasping, pushing, pulling, and rolling without attempting to bite each other. Players usually have a small amount of displacement due to a large range of activities, but they will not move quickly.
	Play biting	Similar to play wrestling but includes players closing their eyes and opening their mouths in a mock attempt to bite each other while at the same time twisting their body to prevent being bitten.
	Play chasing	Locomotor actions such as running, climbing, and leaping towards or away from another individual, in which players alternate their role as chaser and chased. During play chase, there is no physical contact between players.
Affiliative playing	Play mounting	An individual uses its hand to grasp the hair of another player’s body and climbs onto the player’s back. Pelvic thrusting may occur but this does not result in ejaculation. During play mounting, the players are silent. This typically occurs between immature females and immature males.
	Play cuddling	Resembles play wrestling and embracing, with players holding each other with a very slight pushing of their bodies. During play cuddling, the players do not grimace or produce sound. Both parties are in place during the whole process, and the position will not move.
	Play sucking a penis	A player puts his/her playmate’s penis in his/her mouth or actively uses his mouth to approach and hold the playmate’s penis and the playmate occasionally twitches penis. During play, neither player moves, and then when the action is over, the participants continue to play.

**Table 3:** Multiple linear regression for infant Tibetan macaques.

	The Rate of Social Play				The Number of Playmates			
	Infant Males		Infant Females		Infant Males		Infant Females	
	*β*	*p*	*β*	*p*	*β*	*p*	*β*	*p*
Maternal rank	0.247	0.185	−0.455	0.098	0.208	0.277	0.285	0.399
Matriline affiliation/rank	0.249	0.186	0.270	0.253	0.190	0.379	0.011	0.969

**Table 4:** Multiple linear regression for juvenile Tibetan macaques.

	The Rate of Social Play				The Number of Playmates			
	Juvenile Males		Juvenile Females		Juvenile Males		Juvenile Females	
	*β*	*p*	*β*	*p*	*β*	*p*	*β*	*p*
Maternal rank	0.462	0.009	0.797	<0.001	0.189	0.442	0.593	0.020
Matriline affiliation/rank	−0.026	0.865	0.036	0.843	0.143	0.516	−0.111	0.654

## Data Availability

The data that support the findings of this study are available on request from the corresponding author the data are not publicly available due to privacy or ethical restrictions.
